# Functional genomics of complex cancer genomes

**DOI:** 10.1038/s41467-022-33717-8

**Published:** 2022-10-07

**Authors:** Francesca Menghi, Edison T. Liu

**Affiliations:** The Jackson Laboratory for Genomic Medicine, Farmington, CT USA

**Keywords:** Cancer genomics, Cancer genetics

## Abstract

Cancer functional genomics is the study of how genetic, epigenetic, and transcriptional alterations affect cancer phenotypes, such as growth and therapeutic response. Here, we comment on how, taking advantage of next generation sequencing, functional genomics, often combined with systems biology approaches, has revealed novel cancer vulnerabilities beyond the original paradigm of one gene-one phenotype.

## One gene, one phenotype, one drug

For several decades our understanding of the relationship between cancer DNA and cancer biology was based on a finite number of strong cancer genes, whose alterations would lead to the activation of a pro-tumorigenic cellular pathway. The identification of these potent drivers of cellular transformation guided the successful development of the first targeted therapies to antagonize their pro-tumorigenic effect and eventually led to significant improvements in clinical outcomes for cancer patients. Prominent examples of this paradigm are the development of imatinib, a monoclonal antibody targeting the BCR-ABL fusion protein in chronic myelogenous leukemia^1^, of trastuzumab to target HER-2 over-expressing breast cancer patients^2^, and the use of BRAF and MEK inhibitors for the treatment of *BRAF*-mutant melanoma^3^ and of EGFR inhibitors for *EGFR*-mutant lung cancers^4^. Despite these clinical successes, we are approaching a limit to the number of single druggable targets that have clinical efficacy. Not only have we discovered most key drivers for which there are demonstrated tumor dependencies, but most of the cancers treated with single driver therapeutics readily develop drug resistance. Our inability to make further progress in the targeting of individual cancer genes can be attributed to the genomic complexity characterizing most cancers, a complexity that underscores how the concerted activity of multiple genic alterations in a genetically fluid condition orchestrates cancer phenotypic outputs that evolve over time. The relatively modest number of *de facto* cancer drivers with a potential for targeted therapy has been dwarfed by the ever-growing collection of genomic and epigenetic modifications affecting both genes and regulatory elements that, while individually have small to negligible effects, collectively can change the physiology of a cancer through a multitude of paths. Thus, even with actionable mutations, the presence of other activated signaling pathways easily counteracts the effects of targeted interventions (e.g., differences in the impact of *BRAF* V600E mutations in colorectal carcinoma vs. in melanoma^5^). This perspective will focus on our current understanding of how the genome as a whole affects cancer biology, from the description of genome-wide mutational signatures, to GWAS studies that uncover new cancer risk factors, to cellular screening protocols paired with network analysis and systems biology approaches that deconvolute whole genome complexities into specific and actionable cancer dependencies, to the recent discovery of cytosolic fragmented DNA as a pro-inflammatory molecule.

## Interpreting complex genomic profiles

Over the past few years, efforts have been focused on discerning patterns of mutations, i.e., mutational signatures, that accumulate throughout the genome during the process of tumorigenesis. The concept of mutational processes was introduced less than a decade ago, with the identification of specific patterns of somatic single base substitutions across a range of cancer types, which were suggestive of common mechanisms of mutagenesis and potentially ascribable to specific cellular or genetic mechanisms, such as aging, APOBEC activity and homologous recombination deficiency (e.g., mutations in *BRCA1, BRCA2, PALB2, ATR, ATM, RAD51*, etc.); or environmental exposure, including UV light and tobacco^6,7^. For example, UV exposure is associated with mutations at TT dimers, and mutations in DNA mismatch repair genes that underlie Lynch Syndrome (e.g., *MSH2* and *MLH1*) generate InDels in microsatellite repeat tracts throughout the genome. Thus, unlike an individual *RAS* gene mutation that generates a cellular signal that directly affects a cancer phenotype, mutations in these genomic instability drivers induce cancer through subsequent mutations in multiple cancer genes.

Building on these original concepts, subsequent studies aimed to explain how some of the divergent and complex mutational landscapes observed in cancer could have developed from a single initiating event. This led to the discovery of additional forms of mutational signatures and/or genomic scars, including complex rearrangement events such as chromothripsis, chromoplexy and chromoanasynthesis, as well as rearrangement signatures (reviewed in ref. 8). Our own work on the Tandem Duplicator Phenotype (TDP) uncovered a family of genomic instability profiles characterized by hundreds of somatic head-to-tail tandem duplications homogenously dispersed across the chromosomes, many of which systematical perturb combinations of classical oncogenes and tumor suppressors that conjointly drive and sustain tumorigenesis^9^.

The deconvolution of these instability syndromes (e.g., APOBEC, single base substitution signatures, microsatellite instability, TDP) function, in the first instance, as forensic tools for the genomic archeology of cancer. Single base substitution signatures correlate well with specific carcinogen exposures and have led to the identification of a bacterial genotoxin, colibactin, in the mutagenesis of colorectal cancers^10^. Type 1 TDP emerges following conjoint deficiencies in BRCA1 and TP53, microsatellite instability results from the genetic disruption of specific mismatch repair genes (e.g., *MLH1*, *MSH2*, *MSH6*, and *PMS2*). More recently, the utility of these mutational profiles has extended to therapeutics: germline deficiencies in DNA mismatch repair or polymerase proofreading enzymes (such as *POLD1* and *POLE*) give rise to cancers with high tumor mutational burden and that are responsive to immune checkpoint inhibitors (ICIs)^11^. The primary cause of this sensitivity is the generation of neoantigens that render cancer cells more immunogenic than cancers with low mutation rates. This was also found in prostate cancers with disruptive *CDK12* mutations (i.e., TDP group 2/3 mix) that generate fusion neoantigens rendering this otherwise “immunologically cold” cancer type remarkably sensitive to ICI treatment^12^. In these cases, it is no longer mutations in specific genes, but the number and types of coding mutations in many genes that is most critical for therapeutic success.

Another area of genomic complexity is in the germline variations in human populations that can affect cancer risk and cancer outcomes. Although the most dramatic hereditable elements in cancer biology involve actual germline mutations in single susceptibility genes as noted above, GWAS studies have uncovered a number of SNPs associated with increased risk of cancers (reviewed in ref. 13). While these risk factors individually are not useful in clinical predictions, attempts have been made to develop polygenic risk scores (PRS) to aggregate the combinatorial effects of these risk alleles. While it is clear that PRSs correlate with family history, the ability to quantify risk based on the PRS only slightly improves the ability to assess increased risk for cancer. For example, the lifetime risk of developing breast and prostate cancer for individuals assigned to the top 5% of PRS for these diseases increases only from ~12% (i.e., baseline risk) to 19%, and from 13 to 22%, respectively^14^. Two further challenges with PRSs are that, first, the composite risk assessments are based on an additive model and specifically do not take into account gene-gene interactions^15^, or causal proteomic mediators^16^. Therefore, the systems interactions are simply inferred with no knowledge of the contributing components. Moreover, PRSs are ‘tuned’ for the specific populations from which the original GWAS data was derived and are less- or even non-predictive when ported over to other populations^17^. Therefore, while polygenic risk scores estimate whole genome effects, they do not provide the gene-based specificity that other functional and systems genomics approaches have.

## A functional genomic approach to resolve cancer genome dependencies

A great part of gene-based targeted therapeutics is based on exploiting the dependencies of a cancer from specific pathways that drive cell survival and proliferation. However, most cancers present with multiple mutations that result in either overlapping dependencies or bypass mechanisms to overcome these vulnerabilities. With this appreciation of cancer genome complexity, several screening programs have been recently initiated that map cancer cell drug sensitivities to genomic, epigenetic, and transcriptional profiles. Large in scale with respect to both the number of targets/compounds screened and the cancer systems examined, these approaches aim at capturing the high degree of heterogeneity underlying human cancer and to exploit it in an unsupervised manner to uncover specific cancer vulnerabilities that would not be predicted by our current knowledge of cancer biology.

One such initiative is the Cancer Dependency Map (DepMap), an ongoing effort to systematically identify genetic and molecular vulnerabilities across multiple cancer types by integrating CRISPR/Cas9 and shRNA-based genome-wide loss of function screens, small molecule compound screens, and the genomic and transcriptional specificities of hundreds of cancer cell lines^18^. Several cancer cell dependencies have successfully been identified using this approach. For example, Bondeson et al. discovered how overexpression of the phosphate importer SLC34A2, frequently observed in ovarian carcinoma, associates with increased sensitivity to disruption of the XPR1- KIDINS220-dependent mechanism of phosphate efflux, which results in the toxic intracellular accumulation of phosphate and represents a previously unknown therapeutic vulnerability in ovarian carcinoma^19^. The Genomics of Drug Sensitivity in Cancer Project (GDSCP) seeks to identify optimal interventions for specific cancer genetic features by assessing the sensitivity profile of over 1000 genomically and transcriptionally characterized cancer cell lines to a large panel of chemotherapeutic agents and targeted therapies^20^. The NIH library of integrated network-based cellular signatures (LINCS) program focuses on how different genetic and environmental stressors (e.g., growth factors and cytokines) may impact cancer cell pathways and induce cells to switch from a pathogenic to a more physiological state^21^. In each case, these programs integrate the complex somatic genetics of cancers with compendia of interventions or perturbations. Again, the power of the analytical output resides not on the one-to-one relationship between intervention and genomic alterations, but on the discovery of underlying principles of function to craft predictive models.

## Beyond canonical targeted therapeutics: systems biology to unravel complex cancer genome-transcriptome-phenotype associations

We have ascertained that cancer phenotypes are rarely dictated by individual genetic alterations but most commonly by combinations of genomic perturbations. These combinations are complex not only by virtue of numbers, but also because of the heterogeneity of the genetic/transcriptional perturbations: truncating or activating mutations, chimeric fusions, expression changes, splice variants, mutations in regulatory regions, proteomic changes and more. The current challenge is integrating this multitude of changes into cogent, mechanism-based models that can be used to predict biological vulnerabilities and therapeutic possibilities.

The community of systems biologists are addressing these issues of combinatorial complexity by developing new approaches for mapping and modeling cancer pathways through the generation of protein and gene interaction networks (reviewed in Kuenzi et al.^22^). While these approaches differ with respect to how they define functional interactions between proteins and genes (e.g., transcriptional regulation vs. protein-protein interaction), the type of datasets that are integrated and summarized (e.g., gene expression vs. genomics), and the molecular and mechanistic assumptions that they implement (e.g., transcriptional master regulators vs. flux balance), their shared goal is to estimate how specific genomic, epigenetic, transcriptional and/or post-transcriptional contexts translate into differential pathway outputs and ultimately dictate cancer phenotypes and clinical outcomes. To generate meaningful molecular networks, systems biology approaches rely on the availability of large datasets of genomic and functional associations, similar to the ones described above.

More recently, systems biology has been successfully integrated with machine learning approaches to predict precise therapeutic response dependencies. Przedborski et al. described a multi-disciplinary approach combining a well characterized systems biology model of anti-PD-1 immunotherapy to generate simulated clinical trials and a neural network-based classification algorithm that classifies patients based on their therapeutic response^23^. This combined approach allowed to identify biomarkers of anti-PD-1 immunotherapy response in real patients and to speculate on potential mechanisms of drug resistance.

With better genomic datasets arising from comprehensive experimental screening programs, sophisticated systems biology approaches that integrate and interpret them, and structured clinical trials, the two fundamental goals of modern functional genomics—assessing molecular networks, and associating them with specific therapeutic response beyond the canonical targeted therapy candidates—may be achieved simultaneously.

## DNA instability and biochemical response

We have long been working on the premise that DNA mutations contribute to the cancer phenotype because of direct downstream changes in gene activity or protein levels. However, evidence is emerging that defects in homologous recombination deficiency generate cytoplasmic DNA that activates the cGAS/STING pathway leading to production of type 1 interferons and other cytokines^24^. This establishes a pro-inflammatory microenvironment that enhances immune infiltration, and increased sensitivity to TNF-alpha induced cytotoxicity^25,26^. Here, mutations in specific genes are not the inciting factors nor mutations that increase the neoantigen burden, but rather the general increased levels of fragmented DNA from a genomic source.

## The future of functional genomics: challenges and opportunities

The functional genomics of cancer, as we have defined it here, rests either on (1) the complex combinatorial effects of coding mutations in relevant genes that alter cancer phenotypes, (2) the transcriptional cassettes that generate alterations in critical pathways, (3) whole genome mutational signatures that serve as forensic tools to ascertain the origins of a cancer, or (4) whole genome disruption that activate an immune response either by enhancing the neoantigenic load, or activating the cGAS/STING pathway. An emerging complication not discussed thus far is the effect of tumor evolution over time. The ability to evolve and the range of robustness of each cancer progeny against anti-cancer forces also determines whether a tumor can be cured^27,28^. Assessment of such plasticity will need be calculated to complete the full picture of a cancer through its clinical life cycle. Though this has been pursued experimentally^29^ the field is still quite nascent, awaiting more robust methodologies. However, early simulations are already providing a theoretical framework for the evolutionary “steering” of a heterogeneous tumor towards inducing collateral drug sensitivities^30^. Intriguingly, the monitoring of tumor evolutionary dynamics to inform the timing of on/off treatment cycles of anti-androgens in prostate cancer has been applied in the clinic with interesting preliminary results^31^. Therefore, the future is hopeful. Ultimately, it is conceivable that once all regulatory and structural mutations can be detected in a cancer genome with their functions assigned, and the measure of genomic instability ascribed, future computational approaches could better predict the responsiveness and potential curability of cancers even with complex genomes. Equally intriguing is whether targeting the mechanisms that sustain elevated genomic instability may act to limit tumor heterogeneity and to limit the subsequent development of new mutations. Indeed, this may be a new form of adjunctive cancer therapeutics to enhance the curability of genetically complex malignancies.
